# Systemic-to-pulmonary artery shunt: a surgical strategy with no expiration date

**DOI:** 10.3389/fped.2026.1767112

**Published:** 2026-04-21

**Authors:** Luis Emmanuel Ruiz Pérez

**Affiliations:** Deparment of Cardiothoracic Surgery, Hospital ISSSTE Villahermosa, Tabasco, Mexico

**Keywords:** congenital heart disease, palliative cardiac surgery, pulmonary ductal stent, systemic to pulmonary artery shunt, univentricular heart

## Abstract

**Background:**

Systemic-to-pulmonary shunts remain a fundamental surgical option in the palliative management of complex congenital heart defects, particularly in patients with univentricular physiology. Although technically demanding, the procedure has evolved through multiple modifications aimed at simplifying its execution and reducing complications, while maintaining its essential clinical role.

**Objective:**

To provide a narrative review of the Blalock-Taussig-Thomas shunt, examining its physiological basis, surgical technique, associated risks and complications, postoperative management, and historical evolution, with the goal of evaluating its continued relevance in the palliative treatment of complex congenital heart defects.

**Methods:**

A literature search was conducted in Elsevier, PubMed, and Scopus using the keyword “systemic-to-pulmonary shunt.” Studies addressing historical development, technical evolution, clinical indications, and postoperative outcomes were included. The review focused on pediatric populations, specifically prenatal, neonatal, and school-age patients. Studies involving adolescents were excluded to maintain consistency with early developmental stages and the typical clinical context in which these shunts are used.

**Results & conclusion:**

The systemic-to-pulmonary shunt remains a valuable and widely used palliative technique for patients with complex congenital heart defects and univentricular physiology. While the original Blalock-Taussig-Thomas technique has historically shown favorable outcomes, differences in complication rates among its variants are influenced by patient selection and institutional experience. Emerging alternatives, such as ductal stenting, may reduce the frequency of surgical shunt placement in selected patients; however, they do not currently replace the systemic-to-pulmonary shunt as a comprehensive or universally applicable strategy.

## 1 Introduction

Congenital heart defects remain the most common malformations in infancy (1), and although survival has improved significantly—even among patients with complex anomalies (2)—many conditions still require palliative interventions aimed at optimizing pulmonary blood flow. Among these, the systemic-to-pulmonary artery shunt, historically known as the Blalock-Taussig shunt, continues to be one of the most widely used strategies.

The procedure was first performed in 1944 by Alfred Blalock and Helen Taussig (3), with the essential technical contributions of Vivien Thomas, whose role was formally recognized decades later. For this reason, the complete designation—Blalock-Taussig-Thomas shunt—is now widely accepted.

In the 1940s, patients with tetralogy of Fallot faced extremely high mortality rates, as no surgical treatment existed to relieve cyanosis or the consequences of severe hypoxemia. Most died before their first year of life (4). In response to this clinical challenge, Helen Taussig proposed to Alfred Blalock a surgical strategy to redirect systemic blood toward the pulmonary arteries (3), using the subclavian artery as the inflow source to increase pulmonary perfusion, improve oxygenation, and reduce cyanosis.

Based on this principle, a direct arterial connection between the subclavian artery and the pulmonary artery was established (3), resulting in notable clinical improvement. However, complications such as right arm ischemia and, in rare cases, spinal cord injury (5) were reported. In addition, the technique proved challenging in patients with unfavorable anatomy, such as hypoplastic pulmonary artery branches (5), prompting the development of alternative surgical approaches.

**Figure 1:**
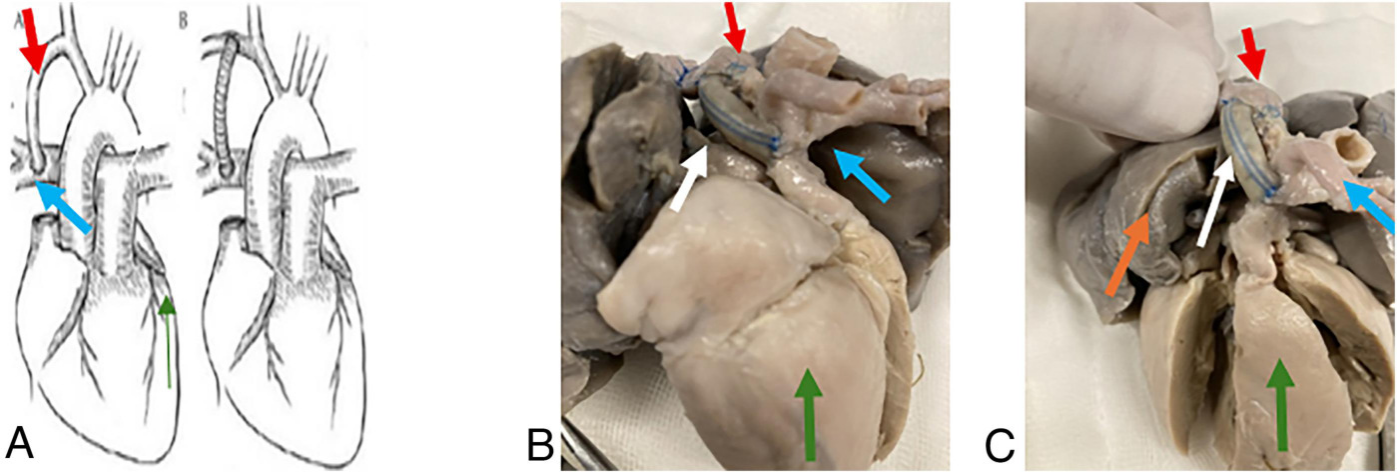
systemic-to-pulmonary shunt consists of a conduit connecting the right subclavian artery to the right pulmonary artery, allowing systemic blood flow to reach the pulmonary circulation. **(A)** Image taken from López-Terrazas JH et al. (12). **(A)** Shows the classic Blalock–Taussig shunt, which connects the left subclavian artery to the pulmonary artery. **(B)** Depicts the contemporary technique, which requires the interposition of a prosthetic conduit between the systemic artery and the pulmonary artery. Red arrow: Right subclavian artery. Blue arrow: Right pulmonary artery. **(B)** Right lateral view of the heart. The image shows how the conduit connects the right subclavian artery to the pulmonary artery. Blue arrow: Pulmonary artery. Red arrow: Right subclavian artery. White arrow: Gore-Tex conduit.Green arrow: Right ventricle. **(C)** Right anterolateral view of the heart. Orange arrow: Right lung. Color coding for the remaining arrows follows the same scheme used in the previous panels.

The systemic-to-pulmonary artery shunt consists of creating a surgical connection between the right subclavian artery and the right pulmonary artery (6) (Figure 1), with the objective of increasing pulmonary blood flow and improving systemic oxygenation. This approach is particularly relevant in patients with univentricular physiology or severe cyanosis. By redirecting systemic blood toward the pulmonary circulation, the shunt promotes pulmonary vascular development and reduces hypoxemia. However, the resulting increase in pulmonary flow also raises venous return to the right atrium, which may predispose patients to pulmonary edema, right-sided heart failure, or pulmonary venous hypertension. For this reason, the systemic-to-pulmonary artery shunt is considered a temporary measure and is typically used as a bridge to subsequent palliative procedures.

One of the earliest modifications was introduced by Klinner, who interposed a prosthetic graft between the subclavian and pulmonary arteries (5) to avoid direct manipulation of the systemic vessel. Other variants sought to bypass the subclavian artery entirely, such as the Potts shunt (descending aorta to the left pulmonary artery) (5) and the Waterston shunt (ascending aorta to the right pulmonary artery) (7, 8). Although these techniques improved cyanosis, they were later associated with pulmonary hypertension and technical difficulties during takedown in subsequent surgeries (6, 7), leading to their decline in contemporary practice.

Other alternatives were also explored, including the use of the internal mammary artery, particularly in patients with congenital absence of the right pulmonary artery (62). However, its surgical complexity – related to its small caliber and limited flow – has prevented its routine use. These limitations supported the continued use of the original concept, refined through the interposition of a prosthetic graft.

Although initially conceived as a palliative treatment for tetralogy of Fallot, the systemic-to-pulmonary artery shunt is now used in a broader spectrum of congenital heart defects with univentricular physiology, including pulmonary atresia, tricuspid atresia, double-outlet right ventricle, and atrioventricular canal defects with a single functional ventricle (9).

Data from the STS Congenital Heart Surgery Database identify this operation among the ten most frequently performed pediatric cardiac surgeries (10), and additional multicenter studies have reported an increase in its use (11), underscoring its ongoing clinical relevance.

This review examines the systemic-to-pulmonary artery shunt with emphasis on surgical technique, risks, complications, postoperative management, and historical evolution, as well as selected technical variants. A systematic comparison of all techniques or a comprehensive management guide is beyond the scope of this article.

## 2 Methods

A literature search was conducted in the Elsevier, PubMed, and Scopus databases using “systemic-to-pulmonary shunt” as the primary keyword, combined with Boolean operators (“AND,” “OR,” “NOT”) and related terms such as “children,” “pediatric,” “complications,” “morbidity,” “other systemic-to-pulmonary shunts,” “ductal stenting,” and similar concepts. Additional terms related to imaging modalities (“magnetic resonance imaging,” “angiography,” “cardiovascular imaging”) were incorporated to increase the sensitivity of the search strategy. The search included publications from 1980 to 2025 and was restricted to articles written in English. Although the search was restricted to English-language publications, one Spanish-language article was included due to the relevance of its anatomical figure for illustrative purposes.

The objective of the search was to identify studies addressing historical development, technical evolution, clinical indications, imaging evaluation, postoperative outcomes, mortality, complications, and medical management. Because certain classic procedures—such as the Potts and Waterston shunts—are no longer performed in contemporary practice, historical and comparative articles were included when they provided essential context for understanding the evolution of systemic-to-pulmonary shunts. Additionally, contemporary studies were incorporated when they referenced or compared these historical techniques. Reference lists of selected articles were manually reviewed, allowing the identification of publications prior to 1980; these were included only when they contributed fundamental information regarding the conceptual or technical development of systemic-to-pulmonary shunts.

Inclusion criteria encompassed original articles—including retrospective studies, prospective studies, case series, cohort studies, database analyses, and comparative studies—as well as review articles, observational studies, and chapters from cardiac surgery textbooks. Studies were included if they involved pediatric populations (neonates, infants, preschool children, school-age children, or adolescents) and addressed systemic-to-pulmonary shunts, ductal stenting, or comparative procedures. Each identified article was initially screened by abstract review, and those meeting relevance criteria were subsequently analyzed in full.

Exclusion criteria included studies that did not provide additional information beyond the selected literature, publications whose full content was not accessible or sufficiently interpretable, and articles not available in English.

## 3 Indications

The main indications for performing a systemic-to-pulmonary artery shunt include severe cyanosis (SaO₂ below 70%–75%) and pulmonary perfusion dependent on a patent ductus arteriosus during the early neonatal period (13). However, any condition—congenital or acquired—that results in restrictive pulmonary blood flow and compromises systemic oxygenation may justify this intervention.

Following placement of the systemic-to-pulmonary artery shunt, the clinical objective is to achieve an arterial oxygen saturation between 75% and 85% (13), provided that diastolic blood pressure remains above 30 mmHg (13). This threshold is essential to ensure adequate cerebral and coronary perfusion, thereby contributing to the patient's hemodynamic stability (13).

## 4 Preoperative management

Patients with congenital heart defects presenting with univentricular physiology may be hemodynamically unstable at birth. Therefore, achieving clinical stability is essential before performing a systemic-to-pulmonary artery shunt (13). In cases of decompensation, initial management may include prostaglandins, inotropic agents, diuretics, and mechanical ventilatory support (13).

Anatomical assessment of the pulmonary vasculature should be performed using echocardiography, and in selected cases, cardiac magnetic resonance imaging may be used to provide a more detailed vascular characterization (14). In patients with unfavorable anatomy, cardiac catheterization allows estimation of the pulmonary vascular bed's capacity and assessment of its tolerance to the increased flow induced by the shunt (15).

## 5 Classical surgical technique

The systemic-to-pulmonary artery shunt is preferably performed during the neonatal period, as the pulmonary vascular bed at this stage is more compliant and better tolerates increased blood Flow (9, 16).

### 5.1 Positioning and surgical approach

The patient is placed in the dorsal decubitus position, with moderate elevation of the corresponding hemithorax depending on the side selected. The surgical approach is made through the third intercostal space, using an incision that extends from the lateral border of the sternum to the axillary line. Entry into the pleural cavity is achieved by dividing the third and fourth costal cartilages (3).

### 5.2 Pulmonary artery preparation

The right pulmonary artery is identified and carefully dissected. Two bulldog clamps are applied: one distal to the bifurcation of the main pulmonary trunk, and another proximal to the branch supplying the upper lobe. A lateral incision is made between both clamps to serve as the site for anastomosis (3).

### 5.3 Systemic vessel preparation

The subclavian or innominate artery is dissected and transected, depending on the case. Intravenous heparin is administered at a dose of 3 mg/kg/day to prevent thrombosis (3). The vessel is isolated using a partial occlusion clamp.

### 5.4 Anastomosis

The end of the systemic vessel is anastomosed to the side of the pulmonary artery using a continuous suture, ensuring proper intima-to-intima alignment. Once the clamps are removed, any bleeding at the suture line is controlled with additional stitches (3).

## 6 Modified technique

This variant was introduced in the early 1960s as an alternative to direct transection of the subclavian artery. Instead of using the native vessel, a synthetic polytetrafluoroethylene (PTFE) graft is employed as a vascular conduit (6). Although this approach preserves the core surgical principles of the original technique (3), it introduces specific modifications in the anastomosis between the subclavian and pulmonary arteries.

### 6.1 Dissection and mobilization

Both the pulmonary artery and the subclavian artery are carefully dissected and mobilized.

### 6.2 Longitudinal incision

A longitudinal incision is made in each vessel, creating an opening that matches the expanded diameter of the graft.

### 6.3 Graft anastomosis

The PTFE graft is anastomosed between the two vessels, allowing blood to flow from the systemic circulation into the pulmonary circuit.

### 6.4 Graft diameter selection based on body weight

Graft diameter selection based on body weight. The diameter of the graft is determined by the patient's body weight, which is why Table 1 is provided. This technique offers greater control over blood flow, reduces direct manipulation of native vessels, and improves the long-term durability of the shunt (6, 16).

**Table 1:** Recommended diameter for systemic-to-pulmonary artery shunt (16).

Weight Category	Recommended Tube Diameter
Neonatos	3.5 mm
5–6 Kg	4 mm
6–10 Kg	5 mm
Over 10 Kg	6 mm

## 7 Purpose of systemic-to-pulmonary shunts

Congenital heart defects with single-ventricle physiology—such as pulmonary atresia, tricuspid atresia, and tetralogy of Fallot—share a structural abnormality that impairs right ventricular outflow to the pulmonary circulation. This obstruction may result from valvular stenosis, atresia, or discontinuity of the outflow tract, leading to a significant reduction in pulmonary blood flow and, consequently, compromised systemic oxygenation.

In the neonatal period, these conditions are further exacerbated by physiological characteristics unique to this stage, including initially elevated pulmonary vascular resistance, immature vascular architecture, and heightened sensitivity to pressure and oxygen fluctuations. These factors render the pulmonary vasculature particularly fragile in the context of congenital defects with restricted pulmonary flow, potentially triggering severe hypoxemia and fatal outcomes if not promptly addressed.

In such patients, pulmonary perfusion may be temporarily sustained by a patent ductus arteriosus (ductus-dependent pulmonary flow). However, this mechanism is often insufficient to ensure adequate oxygenation. For this reason, a systemic-to-pulmonary artery shunt is indicated to augment pulmonary blood flow and support gas exchange.

The goal of this intervention during the neonatal stage is to achieve hemodynamic balance between systemic (QS) and pulmonary (QP) circulation, optimizing the QP/QS ratio. Additionally, it aims to facilitate unobstructed atrial mixing and preserve effective systemic cardiac output.

## 8 Postoperative evaluation

The goal of systemic-to-pulmonary shunts is to improve cyanosis and maintain oxygen saturations between 75%–85% (13), thereby avoiding both hypoxemia and pulmonary overcirculation. Immediate postoperative management focuses on maintaining an appropriate Qp/Qs balance through individualized ventilatory adjustments, including increasing FiO₂ in cases of hypoxemia and using hyperventilation when pulmonary vascular resistance is elevated, as well as implementing fluid restriction and diuretics when there is a risk of volume overload (17). Two of the main risks in patients undergoing systemic-to-pulmonary shunt surgery are hypoxemia and hemodynamic instability; therefore, controlling pulmonary blood flow in the immediate postoperative period is essential to ensure survival and hemodynamic stability (18).

Traditionally, functional assessment of these shunts relied on evaluating oxygen content in pulmonary capillaries after gas exchange (19). However, current practice focuses on two key parameters to assess procedural effectiveness: arterial blood pressure and lactate levels (20). In a prospective observational study, the prognostic value of arterial lactate was evaluated through serial measurements at ICU admission and at 6, 12, and 24 h postoperatively. Patients who died had significantly higher mean lactate levels at all time points (*p* < 0.001) (21). Reported in-hospital mortality was 5.9%, and long-term mortality reached 8.7%, both associated with elevated lactate levels. Hyperlactatemia, defined as lactate >3.0 mmol/L, was identified as an independent predictor of in-hospital mortality (OR = 1.468; 95% CI: 1.239–1.739; *p* < 0.001) and was also associated with an increased risk of long-term mortality (HR = 1.511; 95% CI: 1.251–1.825) (21).

The most commonly used antithrombotic agents after surgery are unfractionated heparin and acetylsalicylic acid; however, the optimal thromboprophylaxis strategy in neonates and children with systemic-to-pulmonary shunts has not yet been established due to variability among centers and the lack of robust comparative evidence (22).

## 9 Imaging evaluation of systemic-to-pulmonary shunts

Currently, less invasive techniques are available to assess the patency of systemic-to-pulmonary shunts. One such method is electrocardiogram-synchronized cardiac magnetic resonance imaging (23), which enables accurate quantification of the pulmonary-to-systemic flow ratio through high-resolution volumetric imaging (24).

Although innovative technologies such as 3D printing and virtual reality visualization have been introduced to enhance the anatomical representation of systemic-to-pulmonary shunts (25), conventional modalities—including biplane angiographic fluoroscopy, rotational angiography, invasive intracardiac echocardiography, and computed tomography—remain fundamental tools in their evaluation. Despite growing interest in these emerging approaches, their clinical application in this context has yet to achieve universal validation (26).

## 10 Main complications

Stenosis and thrombosis are two of the complications most strongly associated with increased mortality in patients with systemic-to-pulmonary shunts (27). A meta-analysis evaluating risk factors in this population reported a global incidence of conduit obstruction of approximately 7% (28). Although conduit size was long considered a potential contributor to mortality, it has since been ruled out as an independent predictor (16, 28). Instead, factors such as patient weight, underlying pathology, coagulation profile, and surgical variables may significantly increase the risk of thrombosis, which is directly linked to higher mortality (28).

Additional conditions that may worsen clinical status in the immediate postoperative period include bradycardia, sepsis, ventilator-associated pneumonia, and heart failure (28).

## 11 Postoperative antithrombotic therapy

Conduit obstruction due to thrombus formation is one of the leading causes of mortality in patients with systemic-to-pulmonary shunts. It has been shown that initiating aspirin therapy within the first 24 h after surgery significantly reduces this risk. Additionally, the combined administration of aspirin and heparin has been associated with higher survival rates compared to heparin alone (29).

Assessing the effectiveness of aspirin remains a challenge, given the limited availability of standardized parameters to accurately quantify its impact. In this context, a retrospective study compared two monitoring methods: thromboelastography with platelet mapping (TPM) and aspirin reaction units (ARU) using the VerifyNow system. The results indicated that ARU monitoring was more efficient, requiring fewer therapeutic adjustments without increasing adverse events, suggesting it may be a more reliable tool for evaluating aspirin sensitivity in pediatric patients (30). However, the study has limitations, including scarce evidence regarding its applicability in children and the fact that not all centers have access to this technology (31).

Despite these limitations, the clinical benefit of aspirin has been supported by multicenter prospective studies, which have demonstrated a significant reduction in the risk of death and thrombosis in pediatric populations (32). A universally accepted pediatric dosage has not been established, and recommendations vary across institutions (33). Some authors propose a range of 1–5 mg/kg/day (34, 35), while an empirical dose of 40 mg/day has been suggested for neonates. In adults, a maximum dose of 75 mg may be sufficient (35). Randomized, double-blind clinical trials have shown that aspirin alone, without clopidogrel, provides comparable efficacy and may offer greater safety in infants with cyanotic congenital heart disease treated with systemic-to-pulmonary shunting (36, 37).

## 12 Comparison between systemic-to-pulmonary shunt and ductal stent placement

Alternative strategies to the systemic-to-pulmonary shunt have been explored with the aim of improving survival in patients with hemodynamic instability. Among these options, ductal stent implantation has emerged as a less invasive alternative to maintain a right-to-left shunt in univentricular hearts with ductus-dependent systemic blood flow. It is indicated in neonates with cyanotic congenital heart disease who remain dependent on ductal patency during the neonatal period (38). However, successful implantation requires ductal diameters between 7 and 9 mm in term neonates to ensure adequate systemic blood flow (39). One of its main advantages is the avoidance of thoracotomy and the complications associated with surgery (38). In addition, this strategy has been shown to improve survival in hemodynamically unstable patients in whom a systemic-to-pulmonary shunt cannot be performed (28, 40).

An observational study reported that patients undergoing ductal stent implantation had a 4.24-fold higher probability of survival compared with those who received a surgical shunt (95% CI: 1.37–13.14; *p* < 0.05) (41). A trend toward reduced use of life-support therapies, such as extracorporeal membrane oxygenation (ECMO), was also observed, with an odds ratio of 0.22 (*p* = 0.058), although this did not reach statistical significance (36).

When comparing clinical outcomes between both groups, ductal stenting was associated with a significantly shorter hospital stay, with an adjusted median difference of 11 days (95% CI: 7.2–14.8; *p* < 0.0001) (42). However, this initial benefit is offset by a higher risk of reintervention: patients with ductal stents were 3.37 times more likely to require a new procedure within the first 3 months (95% CI: 1.91–5.95; *p* < 0.001) and 2.43 times more likely within the first 6 months (95% CI: 1.62–3.64; *p* < 0.001) (42).

Despite these differences, both procedures have proven effective in promoting pulmonary artery growth and improving mid-term survival (43). The choice between the two must be individualized, taking into account anatomy, institutional resources, and available expertise. In very low-birth-weight neonates, those with unfavorable pulmonary artery anatomy, or in centers with limited neonatal interventional cardiology capability, the surgical shunt remains a more predictable strategy with less technical variability. Conversely, in patients with an accessible ductus, favorable anatomy, and available interventional expertise, ductal stenting may offer a less invasive option with faster recovery.

## 13 Discussion

Early complications described with the Blalock-Taussig-Thomas technique included right arm paralysis, attributed to prolonged clamping of the subclavian artery, which caused transient hypoperfusion and spinal cord ischemia (3). To prevent these growth-related complications, Klinner introduced the use of PTFE (polytetrafluoroethylene) vascular grafts between the subclavian artery and the pulmonary artery, which was associated with improved survival (5). As an alternative to conventional techniques, Potts proposed a side-to-side anastomosis between the descending aorta and the left pulmonary artery (5). However, observational studies identified a higher risk of pulmonary hypertension, left ventricular overload, and difficulty reversing the procedure (6), leading to its abandonment.

In recent years, this technique has re-emerged as a palliative option for patients with suprasystemic pulmonary hypertension refractory to medical therapy, demonstrating clinical benefits such as functional improvement, reduced pharmacologic requirements, and a significant decrease in mortality among patients on the transplant waiting list (*p* = 0.02), with notably higher five-year survival rates. Additional reports have documented improved systolic function, reduced afterload, and decreased right ventricular burden (*p* = 0.02) (44, 45), as well as improvement in functional class (46).

Another technical challenge in systemic-to-pulmonary shunts arises in patients with hypoplastic right ventricles and unfavorable pulmonary artery anatomy, where performing a Blalock-Taussig-Thomas shunt becomes technically complex. In this context, the Waterston shunt was introduced as an alternative, consisting of an anastomosis between the ascending aorta and the right pulmonary artery. However, this technique was associated with higher rates of pulmonary hypertension, asymmetric pulmonary artery growth (8), and early mortality (47), limiting its adoption as a first-line surgical strategy (48). Lower-risk variants, such as shunts using the internal mammary artery (44), were developed to simplify the surgical approach, but they fell out of favor due to the structural fragility of the mammary artery (49).

Although multiple variants of systemic-to-pulmonary shunts exist, few studies allow direct comparisons among them. Many analyses have focused solely on identifying factors associated with survival, such as elevated partial pressure of oxygen (PaO₂), which correlates with better outcomes (50). However, there remains a lack of studies comparing survival, surgical feasibility, and complication risk across different shunt techniques (Table 2).

**Table 2:** Comparative characteristics of the main systemic-to-pulmonary shunts.

Characteristics	Classical Blalock–Taussig–Thomas shunt	Modified Blalock–Taussig shunt using a synthetic tube graft	Potts shunt	Waterston shunt
Anatomical site	Subclavian artery to the ipsilateral pulmonary artery	Synthetic conduit between the subclavian artery and the pulmonary artery	Descending aorta to the left pulmonary artery	Ascending aorta to the right pulmonary artery
Flow characteristics	Moderate	Controlled	Very high	High
Complications	Subclavian steal phenomenon	Shunt thrombosis	Severe pulmonary overcirculation	Pulmonary overcirculation
	Upper-extremity ischemia	Shunt stenosis	Pulmonary hypertension	Distortion of the pulmonary artery branches
	Shunt occlusion	Pulmonary artery distortion at the anastomosis site	Congestive heart failure	Progressive pulmonary hypertension
Current clinical use	No longer used in contemporary practice	Current standard of car	Obsolete as a shunt; selectively reintroduced as a reverse Potts shunt for refractory pulmonary hypertension	Obsolete; not used today

The modified systemic-to-pulmonary shunt is the technique most frequently used in pediatric patients. The Potts, classical Blalock–Taussig–Thomas, and Waterston shunts are no longer used in contemporary practice.

Outside these specific contexts, the Blalock-Taussig-Thomas shunt remains a valid palliative option, supported by a one-year survival rate of 82% (95% CI: 72–88.4%) (51). It is considered a safe strategy (52, 53), applicable in infants weighing less than 3 kg, in patients older than 3 years (54, 55), and in those with mildly to moderately elevated pulmonary vascular resistance (56). It has been associated with low operative mortality (2.33%) (55) and significant improvement in oxygen saturation. Long-term follow-up has demonstrated pulmonary artery growth, with an increase in the McGoon index from 0.96 ± 0.48 in the immediate postoperative period to 1.30 ± 0.31 at final evaluation (*p* < 0.05) (54), along with improvement in cardiac functional class and reductions in brain natriuretic peptide levels (57). However, patients presenting with advanced functional class (NYHA III–IV) have shown a higher risk of mortality (HR 5.7; *p* = 0.02) (58).

Several preoperative factors continue to represent significant risks for shunt failure. Multicenter studies have shown that low weight at the time of surgery is associated with a 35% higher likelihood of failure (OR 1.35; *p* = 0.001) (28). However, weight is not the only determinant: the type of shunt also influences outcomes, as ventricle-to-pulmonary artery shunts have a 35% lower risk of failure compared with artery-to-artery shunts (OR 0.65; *p* = 0.020) (59). Contrary to common assumptions, neither conduit diameter nor anastomosis location alone determines long-term success. Recent studies emphasize that precise surgical technique—with proper flow orientation and absence of vascular torsion—is essential to prevent complications such as stenosis, thrombosis, or early shunt dysfunction (16). Even in technically well-executed procedures, adverse events such as progressive stenosis, thrombosis, and pulmonary overcirculation may occur (27). A retrospective study conducted at a pediatric hospital found that patients undergoing systemic-to-pulmonary shunts had an 18.6-fold higher relative risk of overcirculation (95% CI: 3.87–89.4), as well as a 1.57-fold higher risk of obstruction (95% CI: 0.46–5.35), although the latter did not reach statistical significance (*p* < 0.0001) (18).

Comparisons between ductal stenting and surgical systemic-to-pulmonary shunts are currently underway. Observational comparative studies have shown that, in the short term, infants undergoing ductal stent placement have a significantly shorter hospital stay compared with those receiving a surgical shunt (33, 38, 42, 60). However, in the mid- and long-term, ductal stenting is associated with a higher frequency of complications, including stent dysfunction, reduced left pulmonary artery growth, and a higher risk of reintervention compared with surgically managed patients (40, 42).

Consistently, meta-analyses show that ductal stenting may be a non-inferior and even potentially superior strategy to surgical shunts, reporting lower mortality and significantly shorter hospital stays, although with a higher risk of reintervention during follow-up (33). Likewise, recent multicenter studies have reported that ductal stenting is associated with lower inotropic requirements (*p* < 0.001), a higher likelihood of immediate extubation, and shorter duration of mechanical ventilation, again with a higher risk of reinterventions (61). Regarding pulmonary artery growth, meta-analyses have shown comparable outcomes between both strategies in selected patients (33).

Population-based analyses have demonstrated that the risk of reintervention is higher in patients with ductal stents at both 3 months (OR 3.37; 95% CI: 1.91–5.95; *p* < 0.001) and 6 months (OR 2.43; 95% CI: 1.62–3.64; *p* < 0.001), although with significantly lower initial hospital costs compared with surgical shunts32. Nonetheless, appropriate patient selection is essential: those with hemodynamic instability (38) or ductal diameters of 7–9 mm in term neonates benefit the most from this strategy61 (Table 3).

**Table 3:** Comparison between systemic-to-pulmonary shunt and ductal stent placement.

Aspect	Systemic-to-pulmonary Shunt	Ductal stent placement
Indication	Cyanotic congenital heart diseases dependent on systemic-to-pulmonary blood flow	Cyanotic congenital heart diseases dependent on systemic-to-pulmonary blood flow in patients with hemodynamic instability who are not candidates for a systemic-to-pulmonary shunt (38)
Anatomical requirements	Does not depend on ductal diameter; suitable even in the presence of pulmonary artery hypoplasia	Ductus measuring 7–9 mm in term neonates (39)
Patient characteristics	Very low-birth-weight neonates; unfavorable pulmonary artery anatomy; centers without neonatal interventional cardiology capability	Accessible ductus; favorable anatomy; availability of experienced interventional cardiology expertise.
Hospital length of stay	Longer	Shorter
Reinterventions	Lower reintervention rates	Higher rates of reinterventions at 3 and 6 months (42)
Key advantages	More stable flow; extensive surgical experience.	Less invasive; avoids thoracotomy; useful in hemodynamically unstable patients
Key disadvantages	Surgical risk	Anatomical dependence on the ductus; higher reintervention rate
Procedure costs	More expensive	Less expensive

Comparison between systemic-to-pulmonary shunts and ductal stenting, highlighting key indications, advantages, and disadvantages.

## 14 Conclusion

The Blalock–Taussig–Thomas (BTT) shunt remains a contemporary and clinically relevant palliative surgical option in neonates and infants. Although systemic-to-pulmonary shunts have undergone multiple technical modifications, historical variants such as the Potts and Waterston shunts—while informative—were largely abandoned due to complications including pulmonary hypertension, asymmetric pulmonary artery growth, and high early mortality. The modified Blalock–Taussig shunt with an interposed tube continues to be widely used, in part due to its technical familiarity and the favorable safety profile reported across numerous series. Despite good survival in selected populations, shunt-related complications such as thrombosis, stenosis, and pulmonary overcirculation persist, particularly in high-risk patients; therefore, outcomes must be interpreted in the context of institutional experience, patient selection, and surgical era.

The BTT shunt remains a valuable option in the palliative management of congenital heart defects with single-ventricle physiology, especially when alternative strategies are not feasible or carry higher risk. In patients with hemodynamic compromise or contraindications to conventional surgery, ductal stent implantation represents a less invasive alternative with encouraging early results, although evidence regarding long-term outcomes remains limited.

## 15 Limitations

This article aims to provide a narrative overview of the Blalock–Taussig–Thomas systemic-to-pulmonary shunt, addressing its application, management, complications, and comparison with other shunt techniques, without intending to serve as a comprehensive guide. Although the literature on this procedure is relatively abundant, as with many palliative strategies, the quality and strength of the available evidence remain limited. In particular, there is a persistent lack of prospective, multicenter, long-term studies, which hinders the extrapolation of results and the development of evidence-based clinical recommendations. Future research is expected to focus not only on optimizing this technique, but also on evaluating its continued use and the potential for it to be replaced by less invasive alternatives such as ductal stent implantation, as well as exploring its long-term implications for neurodevelopment and quality of life.
